# Distribution of Infections in Patients with Renal Failure Followed in the Intensive Care Unit and the Role of Procalcitonin in Infection Follow-Up

**DOI:** 10.5152/eurasianjmed.2025.25813

**Published:** 2025-06-13

**Authors:** Ferhan Kerget, Edip Erkuş, Buğra Kerget, İbrahim Hakkı Tör

**Affiliations:** 1 Department of Infection Diseases and Clinical Microbiology, Erzurum City Hospital, Erzurum, Türkiye; 2 Department of Nephrology, Health Sciences University, Konya City Hospital, Konya, Türkiye; 3 Department of Pulmonary Diseases, Ataturk University School of Medicine, Yakutiye, Erzurum, Türkiye; 4 Department of Anesthesiology and Reanimation, Health Sciences University, Erzurum Regional Education and Research Hospital, Erzurum, Türkiye

**Keywords:** Acute renal failure, culture, procalcitonin

## Abstract

**Background:**

In this study, the aim was to assess the association between procalcitonin levels and culture positivity in patients with acute renal failure (ARF) admitted to the intensive care unit due to Type 1 and Type 2 respiratory failure.

**Methods:**

About 128 patients with ARF were restrospectively included between January 2022 and December 2023. Based on admission culture results, patients were grouped as infection-positive (n = 40) or infection-negative (n = 88). Laboratory parameters, particularly procalcitonin levels, were compared.

**Results:**

Platelet levels were significantly higher in patients with positive culture results (*P* = .03), while procalcitonin levels did not differ between groups (*P* = .33). Escherichia coli was the most frequently isolated microorganism (25%), with the urinary tract being the most common site of growth. In culture-positive patients, procalcitonin levels exhibited a stronger negative correlation with glomerular filtration rate (GFR) (R = −0.355, *P* = .02) and a positive correlation with creatinine (R = 0.385, *P* = .01), highlighting the impact of renal function. Additionally, procalcitonin levels were positively correlated with C-reactive protein (CRP) (R = 0.586, *P* < .001) and negatively correlated with serum sodium (R = −0.39, *P* = .01) in patients with culture growth. As a secondary observation, platelet levels were elevated in patients with positive cultures.

**Conclusion:**

Although procalcitonin is a recognized marker for infection and sepsis, its diagnostic reliability appears limited in critically ill patients with ARF due to its association with renal dysfunction. Additionally, defining infection based solely on culture positivity has inherent limitations, and further research including comprehensive clinical and laboratory parameters is warranted.

**Main Points**

- Procalcitonin levels were not significantly different between infected and non-infected patients with acute renal failure (ARF), suggesting its limited diagnostic value in this population.
- Platelet levels were significantly higher in patients with culture-proven infections, indicating a potential role for thrombocytosis as an acute-phase reactant in ARF patients.
- *Escherichia coli* was the most frequently isolated microorganism, with urine culture being the most common site of infection.
- Procalcitonin levels showed a negative correlation with glomerular filtration rate and sodium levels, and a positive correlation with C-reactive protein levels.
- Neutrophil count and platelet levels demonstrated better sensitivity and specificity, respectively, in differentiating infected from non-infected ARF patients.

## Introduction

Acute renal failure (ARF) is defined as a sudden and usually reversible decrease in glomerular filtration rate (GFR). This condition leads to an increase in serum blood urea nitrogen, creatinine, and other metabolic waste products normally excreted by the kidneys. Additionally, if urine output is reduced, fluid retention and volume overload may develop.^1^ According to the guidelines published by Kidney Disease Improving Global Outcomes (KDIGO) in 2012, ARF is defined as an increase of 0.3 mg/dL in serum creatinine within 48 hours, a rise of one and a half times the baseline creatinine level within 7 days, or a urine output of less than 0.5 mL/kg/h for 6 hours. The leading causes of mortality in patients with ARF are cardiovascular events and infections.^2^

ARF is more frequently encountered in hospitalized patients than in the general population. While the incidence of community-acquired ARF is approximately 1%, the prevalence rises to 4.9-7.2% in hospitalized patients and reaches around 30% in intensive care units, with prerenal causes being the most common etiology.^3,4^ Renal dysfunction in patients with ARF may also weaken immune responses, predisposing these patients to infections. However, there is no consensus on which biomarkers and cut-off values should be used to reliably detect infections in patients with impaired renal function.^5^ Despite this uncertainty, C-reactive protein (CRP) and procalcitonin are among the most commonly used parameters in clinical follow-up.

Procalcitonin is the prohormone of calcitonin and is known to increase predominantly in bacterial infections and sepsis as part of the systemic inflammatory response. It generally does not rise in viral infections; however, elevated cytokine levels may occasionally lead to increased procalcitonin levels. In patients with renal failure, procalcitonin clearance decreases in parallel with the decline in GFR, resulting in higher-than-expected serum levels. Therefore, the use of procalcitonin as an infection marker in patients with ARF, particularly when serum creatinine levels exceed 2 mg/dL, remains controversial.^6^^,^^7^ Additionally, both procalcitonin and CRP are partially cleared by renal replacement therapies, further limiting their reliability as infection biomarkers in this patient population.^8^

In this study, the aim was to evaluate whether procalcitonin levels are associated with culture positivity and to determine the distribution of infectious agents in critically ill patients with ARF and hypoxic respiratory failure. This study focuses on patients with both ARF and hypoxic respiratory failure, and the potential independent impact of respiratory failure on procalcitonin levels could not be evaluated due to the lack of a comparative control group.

## Materials and Methods

### Study Design

A total of 128 patients aged 18 years and older who were diagnosed with ARF and followed up with hypoxic respiratory failure in intensive care between January 2022 and December 2023 were included in the study. Acute renal failure was diagnosed according to the Kidney Disease: Improving Global Outcomes (KDIGO) guideline. According to KDIGO, the definition and staging of acute kidney injury (AKI) are as follows: AKI was defined as an increase in serum creatinine of 0.3 mg/dL within 48 hours, or one and a half times above the baseline creatinine value within 7 days, or a urine output of less than 0.5 mL/kg/h for 6 hours. Patient records were reviewed retrospectively. Before starting the study, approval was obtained from Erzurum City Hospital’s local ethics committee (Approval No:BAEK 2024/08-153, Date: 14.08.2024), and written informed consent was obtained from all patients included in the study.

### Study Group

Blood, urine, and sputum cultures were obtained at the time of admission for patients admitted to the hospital’s intensive care unit due to hypoxic respiratory failure. According to the culture results taken at admission, patients were divided into 2 groups after excluding results considered to be contaminated. Growth was observed in at least 1 culture in 40 patients, while no growth was detected in any cultures in 88 patients. Laboratory parameters were checked at the time of culture sampling and recorded. Infection was defined solely based on positive culture results obtained at admission. Clinical parameters such as Sequential Organ Failure Assessment (SOFA) score, CRP, and white blood cell (WBC) count were not used to define infection status in this study.

### Exclusion Criteria

Patients who had received antibiotic therapy for any reason within the last month, those who were followed up for acute myocardial infarction or acute coronary events within the previous month, patients with malignancy, patients with chronic renal failure, those diagnosed with primary or secondary thrombocytosis, patients who had undergone thyroid surgery, and patients who had been hospitalized within the last month were excluded from the study.

### Definition of Hypoxic Respiratory Failure

Hypoxic respiratory failure is classified into 4 types. In this study, patients with Type 1 and Type 2 respiratory failure who presented with shortness of breath and were admitted to intensive care with predominant obstructive or restrictive pulmonary pathology were included. Type 1 respiratory failure was defined as a PaO2 level below 60 mmHg, while Type 2 respiratory failure was defined as a PaCO2 level above 45 mmHg in arterial blood gas analysis. This study was designed as a retrospective observational analysis comparing patients with culture-positive results to those without growth; no control group of ARF patients without respiratory failure or intensive care unit patients without ARF was included.

### Statistical Analysis

Analyses were performed using the IBM SPSS 20 (IBM SPSS Corp.; Armonk, NY, USA) statistical analysis program. Data were presented as mean, SD, median, minimum, maximum, percentage, and number. Shapiro Wilk-W and Kolmogorov Smirnov tests were used to check the normal distribution of continuous variables. In comparisons between 2 independent groups, the Independent Samples t-test was used when the normal distribution condition was met, and the Mann-Whitney U test was used when it was not met. In 2 × 2 comparisons between categorical variables, if the expected value (>5) was determined, the Pearson Chi-square test was used; if the expected value (3-5) was determined, the Chi-square Yates test was used, and if the predicted value (<3) was determined, Fisher’s Exact test was used. Spearman correlation was used in the correlation analysis of creatine, GFR, CRP, and sodium levels. Receiver operating characteristic (ROC) analysis was used in the sensitivity and specificity analysis of laboratory, age, and hospital stay between patients with and without growth. The statistical significance level was taken as *P* < .05.

## Results

The mean age of the patients included in the study was 68.9 ± 14.9. The mean age of the patients with positive culture was 72.4 ± 14.2, while the mean age of the patients with a negative culture was 67.2 ± 14.9. Fifty-two of the patients included in the study were male (40.6%). Nineteen of the patients with positive culture were male (47.5%). No statistically significant difference was observed when comparing the patients according to age and gender (*P* = .06, .36, .74, respectively).

Diabetes mellitus (DM) was observed in 51 patients, and hypertension (HT) in 67 patients. Culture growth was observed in 16 (31.4%) of the DM patients, while culture growth was observed in 20 (29.9%) of the HT patients. No significant difference was observed when comparing comorbidities between the groups (*P* = .89, .53, respectively).

Table 1 compares the age, hospital stay, and laboratory findings of patients with and without culture growth. Accordingly, platelet levels were statistically significant and higher in patients with culture growth than in those without (*P* = .03).

**Figure 1.**
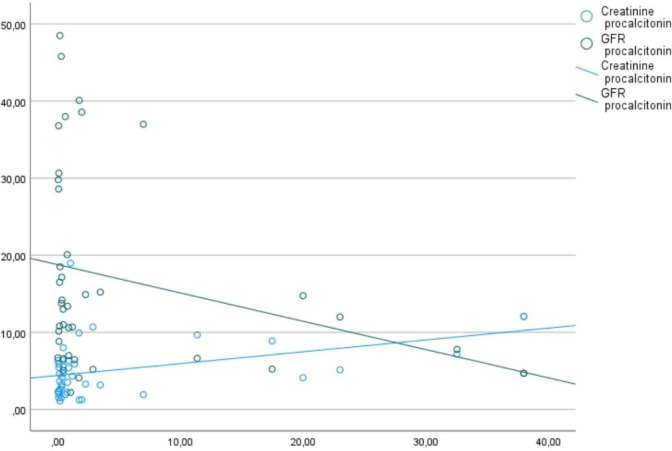
Analysis of the correlation between procalcitonin, glomerular filtration rate, and creatinine levels in patients with positive cultures.

**Figure 2.**
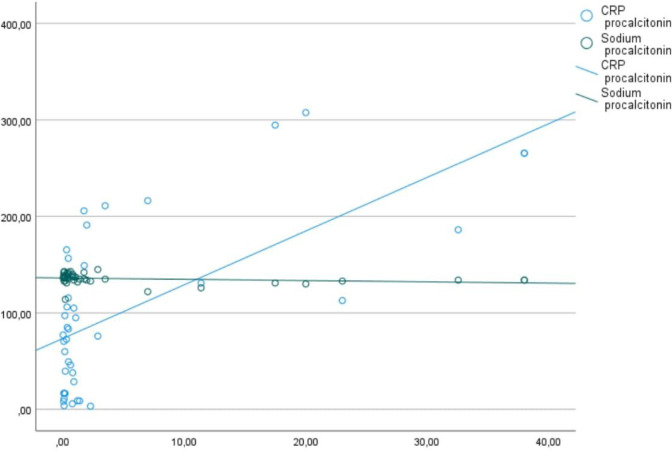
Correlation analysis of procalcitonin with C-reactive protein and sodium levels in patients with positive cultures.

Table 2 shows the types of bacteria grown in the culture and their growth sites. Accordingly, *Escherichia coli* was the most frequently observed bacteria, with 25%. *Acinetobacter boumanii, Stenotrophomonas maltophilia, Enterococcus feacalis + Klebsiella pneumonia, Proteus mirabilis, *and* Pseudomonas aeruginosa* were observed in only 1 patient each. The correlation analysis of GFR and creatinine levels with procalcitonin levels in patients showing culture growth is illustrated in Figure 1. Additionally, the correlation analysis of CRP and sodium levels with procalcitonin levels is depicted in Figure 2. In the correlation analysis of the patient’s laboratory data, it was observed that procalcitonin level was negatively correlated with GFR level and positively correlated with creatinine level (R = −0.355, *P* = .02, R = 0.385, *P* = .01, respectively). It was also observed that procalcitonin level was positively correlated with CRP level and negatively correlated with serum sodium level (R = 0.586, *P* = <.001, R = −0.39, *P* = .01, respectively). In the correlation analysis of patients with no culture growth, it was observed that only the procalcitonin level and the CRP level were correlated (R = 0.473, *P* < .001).

**Figure 3.**
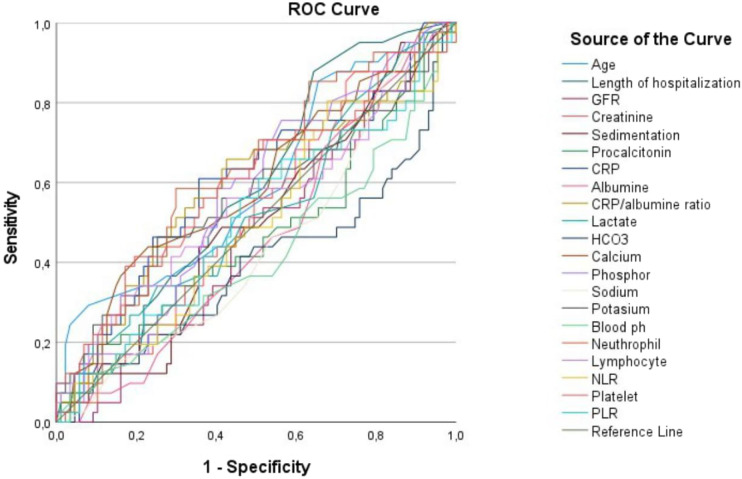
Receiver operating characteristic curve analysis of age, hospital stay, and laboratory parameters in patients with and without culture growth.

The ROC analysis of laboratory parameters, age, and hospitalization period between patients with and without culture growth is shown in Figure 3. Accordingly, it was observed that only neutrophil and platelet levels showed a statistically significant difference in discrimination. When the cut-off value for neutrophil count was taken as 8240/µL, the sensitivity was observed as 60% and the specificity as 70% in this discrimination. When the cut-off value for platelet level was taken as 256 500 (/L), the sensitivity was observed as 42% and the specificity as 80% (Table 3).

## Discussion

Our study observed that platelet levels were higher in patients with ARF and culture growth during intensive care admission than in patients without growth. The most common cause of infection was *Escherichia coli* *(E. coli),* and urine culture positivity was observed more frequently. No relationship was shown between procalcitonin levels and culture growth, but an inverse correlation was observed with GFR. In addition, sodium levels were lower in patients with high procalcitonin levels. In the ROC analysis of demographic and laboratory parameters in patients with and without growth, it was observed that the sensitivity of neutrophil levels and the specificity of platelet levels were better in differentiating between patients with and without growth.

Cardiovascular and infectious diseases are the most common complications in patients with renal dysfunction. According to the 2024 US Renal Data System report, ARF was determined to be the most common cause of hospitalizations accompanied by sepsis.^9^ In the 2022 data, 49.1% of hospitalized patients with ARF had sepsis, 26.1% had non-sepsis infections, and 19.5% had cancer-related causes. The most common AKI is sepsis, with a rate of 15.2%.^10^ Urinary system infections are the most common in community-acquired infections, and *E*.* coli* is the most common microbial agent.^11^^,^^11^In this study, urinary system infection was the most common, and *E. coli* was isolated as the most common agent, consistent with the literature.

Thrombocytosis or thrombocythemia occurs when the platelet count exceeds 450 000/μL and is classified as primary or secondary thrombocytosis. Secondary thrombocytosis is reactive thrombocytosis triggered by conditions such as infections, inflammation, bleeding, or certain medications, without chronic myeloproliferative disease. In secondary thrombocytosis, inflammatory compounds (e.g., IL-6) increase thrombopoietin secretion and subsequent megakaryocyte production, increasing platelet count.^12^Acute bacterial, viral, or chronic infections (e.g., tuberculosis) may cause secondary thrombocytosis by increasing thrombopoietin synthesis through increased inflammatory component IL-6. Studies have identified infectious agents in 75% of pediatric patients with reactive thrombocytosis.^14^^-^^15^Inflammation promotes proplatelet production and hepatic thrombopoietin synthesis in megakaryocytes by increasing IL-6 secretion, possibly leading to thrombocytosis and potential thrombosis.^17^^-^19 In this study, in line with the literature, platelet levels were higher in patients with culture growth and proven infection compared to patients without growth. These findings suggest that platelet levels can be used as a reliable acute-phase reactant in detecting and following up infection in patients with renal failure.

Procalcitonin (PCT) levels can be challenging to interpret in patients with comorbidities because conditions such as massive stress, medullary thyroid carcinoma, and small-cell lung cancer can elevate PCT levels even in the absence of bacterial infection.^20^ PCT levels are also frequently elevated in patients with renal dysfunction in the absence of disease.^21^ Interestingly, the PCT half-life is similar in patients with and without renal dysfunction, approximately 28.9 hours in those without renal dysfunction and 33 hours in those with dysfunction. However, PCT levels in uninfected patients with renal dysfunction are significantly higher than the threshold value of <0.1 ng/mL in healthy individuals.^22^The use of PCT as a marker of infection is made difficult by the high baseline PCT values in patients with renal dysfunction. Limited studies have determined the optimal PCT threshold for the diagnosis of infection in patients with renal dysfunction. The literature reports that PCT levels are elevated in patients with noninfected renal dysfunction.^21^^,^^23^ The study found that the mean PCT in noninfected patients was as high as 3.5 ng/mL. As a result, it was determined that PCT is not reliable for distinguishing infection in ARF patients when evaluated solely by culture positivity without clinical infection scoring.

An additional consideration is that patients included in this study were selected based on admission to intensive care due to type 1 or type 2 respiratory failure. The influence of respiratory failure on infection risk, inflammatory response, and procalcitonin dynamics was not specifically analyzed and may affect the interpretation of the findings.

This study also observed that procalcitonin levels were negatively correlated with GFR and sodium levels and positively correlated with CRP levels, particularly in patients with culture growth. Renal damage in patients with significant GFR impairment may have increased acute-phase reactant CRP independently of infection, while decreased procalcitonin clearance due to renal dysfunction may have contributed to elevated levels. The observed negative correlation with sodium levels may reflect the presence of hyponatremia associated with renal dysfunction and systemic inflammation. Furthermore, among all the parameters investigated, there was a significant difference in the sensitivity of neutrophils and the specificity of platelets in distinguishing infected individuals from non-infected individuals. In the acute inflammatory process, elevated neutrophil levels and reactive thrombocytosis appear to be important indicators. It was believed that the combined evaluation of neutrophil count and thrombocytosis can be effective in differentiating infection status in patients with ARF.

This study was conducted based on culture results obtained during hospitalization, and culture results obtained 48 hours after hospitalization were not included. Intensive care units are suitable environments for coinfections, and this method was chosen to prevent situations that could affect patients’ results. However, studies confirmed by repeated culture results in an environment where coinfections can be prevented could contribute more to the literature.

In conclusion, while procalcitonin is commonly used in diagnosing and following up on bacterial infection and sepsis, its diagnostic reliability in patients with ARF remains uncertain when evaluated against culture positivity alone. Larger prospective studies incorporating clinical infection scores and broader infection definitions are required to determine its true diagnostic value in this population.

## Figures and Tables

**Table 1.** Comparison of Age, Hospitalization, and Laboratory Values of Patients with and Without Culture Growth

Category	Parameter	Culture Positive (n = 40) Mean ± SD	Culture Negative (n = 88) Mean ± SD	*P*
**Demographics**	**Age (years)**	72.4 ± 14.2	67.2 ± 14.9	.06
	**Length of hospitalization (days)**	17.2 ± 12.3	15.1 ± 11.2	.32
**Renal function**	**GFR (mL/min/1.73 m²)**	16.9 ± 12.9	19.9 ± 17	.31
	**Creatinine (mg/dL)**	5.2 ± 3.7	5.1 ± 3.7	.88
	**Phosphor (mg/dL)**	5.8 ± 2.8	5.1 ± 2.4	.31
	**Blood pH**	7.31 ± 0.11	7.4 ± 0.9	.38
**Inflammatory markers**	**Procalcitonin (µg/L)**	5.2 ± 10.4	3.5 ± 7.4	.33
	**CRP (mg/dL)**	102.5 ± 87.1	79.5 ± 87.5	.16
	**CRP/Albumin ratio**	4.9 ± 9.8	3.6 ± 10.4	.48
	**Sedimentation (mm/h)**	27.2 ± 20.9	27.6 ± 20.8	.91
**Hematologic parameters**	**Neutrophil (/µL)**	8817.8 ± 4427.7	7898.2 ± 6251.7	.39
	**Lymphocyte (/µL)**	1582.2 ± 2595.1	999.3 ± 610.1	.16
	**Platelet (/µL)**	240 219.5 ± 109 819.5	201 137.9 ± 83 891.8	.03
	**NLR**	11.3 ± 11.3	11.4 ± 11.3	.98
	**PLR**	302.6 ± 265.8	301.5 ± 282.9	.98
**Electrolytes and others**	**Sodium (mmol/L)**	135.5 ± 5.7	135.3 ± 15.7	.91
	**Potassium (mmol/L)**	4.8 ± 1.3	4.4 ± 0.9	.09
	**Calcium (mg/dL)**	8.4 ± 1.2	8.1 ± 1.1	.16
	**HCO₃ (mEq/L)**	17.7 ± 5.4	19.4 ± 4.7	.09
	**Lactate (mmol/L)**	2.3 ± 1.7	2.1 ± 1.4	.36

CRP, C-reactive protein; GFR, glomerular filtration rate; PLR, platelet-lymphocyte ratio.

**Table 2.** Bacteria Grown in Culture and Their Growth Sites

	Bacteria Type on Reproduction Culture (n/%)	
*MRSA*	5 (12.5%)	Blood
*Escherichia coli*	12 (25%)	Urine
*Klebsiella pneumonia*	2 (5%)	Urine
*Candida parapsilosis*	2 (5%)	Urine
*Candida alcicans*	4 (10%)	Urine
*Enterococcus feacalis*	1 (2.5%)	Urine
*Pseudomonas *spp.	5 (12.5%)	2 blood, 3 urine
*Acinetobacter boumanii*	1 (2.5%)	Urine
*Stenotrophomonas maltophilia*	1 (2.5%)	Blood
*Enterococcus feacalis + Klebsiella pneumonia*	1 (2.5%)	Both in blood and urine
*MRKNS + Klebsiella pneumonia*	2 (5%)	Both in blood and urine
*Streptococcus pneumonia*	2 (5%)	Blood
*Proteus mirabilis*	1 (2.5%)	Blood
*Pseudomonas aeruginosa*	1 (2.5%)	Blood

**Table 3.** Receiver Operating Characteristic Curve Analysis of Neutrophil, Platelet, and Procalcitonin Levels Between Patients with and Without Culture Growth

Area Under the Curve					
Test Result Variables	Area	Std. Error	*P*	Asymptotic 95% CI	
				Lower Bound	Upper Bound
**Neuthrophil (/µL)**	0.617	0.053	.03	0.513	0.720
**Platelet (/µL)**	0.604	0.056	.05	0.495	0.714
**Procalcitonin (µg/L)**	0.526	0.058	.64	0.413	0.635

## Data Availability

The data that support the findings of this study are available on request from the corresponding author.
